# Temporal and spatial analysis of over 7,000 measles cases outbreak from 2018 to 2019 in the Brazilian Amazon

**DOI:** 10.31744/einstein_journal/2024AO0931

**Published:** 2024-03-19

**Authors:** Felipe de Mello Martins, Alessandra Pinheiro Vidal, Jeevan Giddaluru, Bernardo Maia da Silva, Eva K. Lee, Peijue Zhang, Lucas Esteves Cardozo, Carlos Augusto Prete, Helves Humberto Domingues, Ester Cerdeira Sabino, Vanderson de Souza Sampaio, Wuelton Marcelo Monteiro, Helder I Nakaya

**Affiliations:** 1 Universidade do Estado do Amazonas Escola Superior de Ciências da Saúde Manaus AM Brazil Escola Superior de Ciências da Saúde, Universidade do Estado do Amazonas, Manaus, AM, Brazil.; 2 Fundação de Medicina Tropical Doutor Heitor Vieira Dourado Manaus AM Brazil Fundação de Medicina Tropical Doutor Heitor Vieira Dourado, Manaus, AM, Brazil.; 3 Universidade de São Paulo Faculdade de Ciências Farmacêuticas São Paulo SP Brazil Faculdade de Ciências Farmacêuticas, Universidade de São Paulo, São Paulo, SP, Brazil.; 4 Georgia Institute of Technology Center for Operations Research in Medicine and Healthcare Atlanta USA Center for Operations Research in Medicine and Healthcare, Georgia Institute of Technology, Atlanta, USA.; 5 Universidade de São Paulo Escola Politécnica Department of Electronic Systems Engineering São Paulo SP Brazil Department of Electronic Systems Engineering, Escola Politécnica, Universidade de São Paulo, São Paulo, SP, Brazil.; 6 Universidade de São Paulo Instituto de Medicinal Tropical Faculdade de Medicina São Paulo SP Brazil Instituto de Medicinal Tropical, Faculdade de Medicina, Universidade de São Paulo, São Paulo, SP, Brazil.; 7 Rosemary Costa Pinto Fundação de Vigilância em Saúde do Amazonas Dra Manaus AM Brazil Fundação de Vigilância em Saúde do Amazonas Dra. Rosemary Costa Pinto, Manaus, AM, Brazil.; 8 Instituto Todos pela Saúde São Paulo SP Brazil Instituto Todos pela Saúde, São Paulo, SP, Brazil.; 9 Hospital Israelita Albert Einstein São Paulo SP Brazil Hospital Israelita Albert Einstein, São Paulo, SP, Brazil.

**Keywords:** Measles, Disease outbreaks, Vaccination, Geographic information systems, Spatial analysis, Age distribution, Prevalence

## Abstract

**Objective:**

This study aimed to present a temporal and spatial analysis of the 2018 measles outbreak in Brazil, particularly in the metropolitan city of Manaus in the Amazon region, and further introduce a new tool for spatial analysis.

**Methods:**

We analyzed the geographical data of the residences of over 7,000 individuals with measles in Manaus during 2018 and 2019. Spatial and temporal analyses were conducted to characterize various aspects of the outbreak, including the onset and prevalence of symptoms, demographics, and vaccination status. A visualization tool was also constructed to display the geographical and temporal distribution of the reported measles cases.

**Results:**

Approximately 95% of the included participants had not received vaccination within the past decade. Heterogeneity was observed across all facets of the outbreak, including variations in the incubation period and symptom presentation. Age distribution exhibited two peaks, occurring at one year and 18 years of age, and the potential implications of this distribution on predictive analysis were discussed. Additionally, spatial analysis revealed that areas with the highest case densities tended to have the lowest standard of living.

**Conclusion:**

Understanding the spatial and temporal spread of measles outbreaks provides insights for decision-making regarding measures to mitigate future epidemics.

## In Brief

Martins et al. extensively investigated the 2018 measles outbreak in Manaus, Brazil, by integrating visual analytics with traditional epidemiology. They found that most cases occurred in unvaccinated individuals and highlighted the influence of socioeconomic factors on the spread. This study highlighted the importance of visual tools in the development of robust strategies for health emergencies.

## Highlights

▪ Approximately 95% of the measles cases reported occurred in unvaccinated individuals.
▪ Our findings revealed that socioeconomic factors influence the spread of the disease.
▪ Visual analytics enhanced epidemiological analysis.

## Graphical abstract

**Figure:**
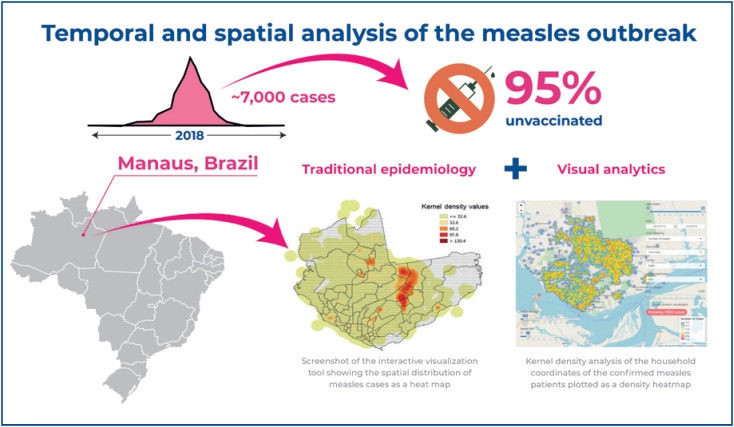

## INTRODUCTION

Measles is a highly infectious acute febrile illness caused by the measles virus. Prior to extensive vaccination in the 1980s, this disease claimed the lives of millions annually and was estimated to cause approximately 128,000 deaths in 2021.^(1)^ The virus is primarily transmitted through respiratory droplets from infected individuals during coughing and sneezing.^(2)^ However, transmission can also occur through inhalation of air contaminated with the measles virus or by touching an infected surface and subsequently touching the mouth, nose, or eyes.^(2)^

Symptoms of measles typically include fever and at least one of the following: cough, coryza, or conjunctivitis. Additionally, a characteristic erythematous maculopapular rash, also called exanthema, typically develops after the onset of early symptoms; however, the timing of this symptom varies considerably.^(2)^ Complications from measles are most prevalent among specific demographic groups, notably children under the age of two, adults over the age of 20, pregnant women, and malnourished or immunocompromised individuals, especially children.

Vaccination against measles is crucial in preventing infection, as approximately 90% of non-immune individuals in close contact with someone with measles may become infected.^(3)^ With an effectiveness of at least 95%, the measles vaccine has been instrumental in preventing outbreaks in many countries and has saved several million lives globally.^(3)^ In 2016, after decades of measles control and more than six years without any reported endemic cases, Brazil received a "certificate of measles elimination" from the Pan American Health Organization (PAHO).^(4)^ However, an outbreak of almost 8,000 potential measles cases emerged in the Brazilian Amazon region in 2018.^(5)^ This outbreak subsequently spread throughout the country, resulting in 21,901 cases nationwide in 2019 and 8,448 confirmed cases in 2020. Furthermore, despite extensive vaccination campaigns, Brazil still recorded 19 laboratory-confirmed cases, with another 217 suspected cases under investigation in the first four months of 2022.^(6)^ Consequently, Brazil lost its elimination status in April 2019 due to the reestablishment of endemic transmission.

Epidemiological surveillance is vital to achieving and maintaining the elimination of measles. It involves promptly identifying and reporting suspected cases within the population and implementing relevant prevention and control measures. Upon detecting a suspected case (*i.e*., the index case), health authorities are mandated by the World Health Organization (WHO) to perform contact tracing and to either vaccinate or isolate any non-immune person who may have been exposed to the virus.^(5)^

Herein, we provide a detailed and comprehensive characterization of the recent measles outbreak in Manaus using the latest available data. The characteristics of the outbreak were described using statistical modeling and spatial analyses.

## OBJECTIVE

This study aimed to present a temporal and spatial analysis of a measles outbreak and introduce a new tool for spatial analysis.

## METHODS

### Data collection

In the northern region of Brazil, specifically in the State of Amazonas, the city of Manaus is home to over 2.1 million residents. The first notification of the measles outbreak occurred on February 21, 2018. Subsequently, the number of suspected cases rose in the following weeks before experiencing a sharp decline by late October of the same year, and the most recent case included in our analysis was reported on March 3, 2019. In Brazil, it is mandatory for all suspected cases of measles to be reported to local sanitary authorities, with further registration in the National System of Notifiable Diseases. Epidemiological and laboratory investigations, as well as contact tracing, were conducted by healthcare professionals at municipal, state, and federal levels. Suspected cases were directly reported to the Brazilian Ministry of Health via an electronic system using a standardized form created by the federal government for reporting both measles and rubella cases. This form included details such as the patient's address, dates of symptom onset and treatment, and vaccination status, as provided by the individual. The form is presented in Supplementary Material. An epidemiological link denotes a case associated with another laboratory-confirmed case, with the connection established if any contact between the suspected case and the laboratory-confirmed case occurred within the month prior to the appearance of the rash. Diagnosis was made through standard immunoglobulin (Ig)M/IgG serology and seroconversion. Positive cases were defined as those testing positive for IgM/IgG and exhibiting seroconversion in a second sample collected 15 – 25 days after the first sample. A flowchart outlining the case determination process is presented in figure 1S (Supplementary Material). We retrieved this data from the Ministry of Health and converted them into comma-separated value files for analysis.

### Spatial analyses

Spatial analyses were performed using the geographical home coordinates of all patients with measles between 2018 and 2019. The spatial mean center of all home coordinates was determined at the start. The mean center represents the average location of all geographical data points. Subsequently, the spatial standard distance was computed to measure the dispersion of each feature point from the mean center (Figure 2S, Supplementary Material). Finally, the standard distance was utilized as an input parameter to perform Kernel density estimation (KDE) using the Gaussian approximation.^(7)^ This facilitated the calculation of the optimum bandwidth and the creation of a density heatmap. All analyses were performed using QGIS 3.8.2 software, utilizing the SAD69(96)/Brazil Polyconic ESPG: 5530 projected coordinate system with meters as the unit of measurement.

### Epidemiological modeling

Mathematical models of infectious diseases play a vital role in transforming available information into insights regarding disease propagation.^(8)^ In this study, an ordinary differential equation-based compartmental susceptible-infectious-recovered (SIR) disease propagation model was used to analyze the measles outbreak. The SIR model categorizes the population into three basic compartments: the susceptible population (S), the infectious population (I), and those who have recovered from the illness (R). Instead of utilizing the Susceptible-Exposed-Infectious-Recovered (SEIR) model, the SIR model is selected to prevent potential overparameterization due to limited data availability.

Notably, a substantial proportion of the population is often immune to measles because of proper preventive vaccination. Specifically, it was reported that between 2015 and October 2018, Measles-Mumps-Rubella (MMR) vaccine coverage in Brazil dropped from 96.1% to 86.7% and subsequently increased to 95% after the national vaccination campaign in September 2018.^(9,10)^ We excluded these vaccinated (immune) individuals from the SIR model since they do not transition between population compartments during the spread of the disease.

In the SEIR model, individuals transition between compartments based on factors such as the timing of exposure, the infective period, the contact rate, and the transition probability matrix. Transmission parameters were estimated using 2018 measles data. Additionally, to simulate intervention scenarios (*e.g*., public campaigns for isolation and catch-up vaccination), a host resistance factor was introduced to capture intervention effects. This involved dynamically adjusting the contact rate to reflect changes in situational awareness (e.g., through social media news). Figure 3S, Supplementary Material demonstrates an intervention scenario where the contact rate was reduced by 20% two months after its initiation. The complete model and parameters used for our analysis are summarized in the Supplementary Material. Given that measles primarily affects unvaccinated children, the susceptible population was set to 3.9% – 13.3% of the total population (corresponding to the range of the unvaccinated population).

### Visualization online tool

An interactive visualization tool for the spatial distribution of measles cases was developed using R packages Shiny and Leaflet. To safeguard data confidentiality, uniformly distributed noise with a maximum amplitude of 100 m was added to the geographical coordinates of each address. The tool allows the visualization of noisy coordinates as circles scattered across the map, grouped in clusters, or as a heat map. Moreover, the data can be filtered by date, sex, age, and final classification (laboratory-confirmed or clinically confirmed).

### Ethical aspects

The Research Ethics Committee of *Fundação de Medicina Tropical Doutor Heitor Vieira Dourado* approved the study under CAAE: 93002318.10000.0005; # 2.903.504.

### Role of the funding source

The funders played no role in the study design, data collection, data analysis, interpretation, or writing of the report.

## RESULTS

A summary of the measles outbreaks is presented in figure 1. Among the 9,790 suspected cases reported to the Ministry of Health, 7,896 were confirmed as measles based on clinical or laboratory findings (Figure 1A). The outbreak occurred between May and September 2018, at the peak of the Amazonian dry season (Figure 1C). Of the confirmed measles cases, 94.9% (7,492) of the individuals were either never vaccinated or had received a vaccination against measles a decade ago before the disease (Figure 1B). The remaining 5.1% (404 patients) had received the vaccine less than ten years before diagnosis; this subgroup comprised 215 patients who had been vaccinated over a year previously, 30 who had received the vaccine between six months and a year previously, and 159 who had received it between two and six months previously (Figure 1C).

**Figure 1:**
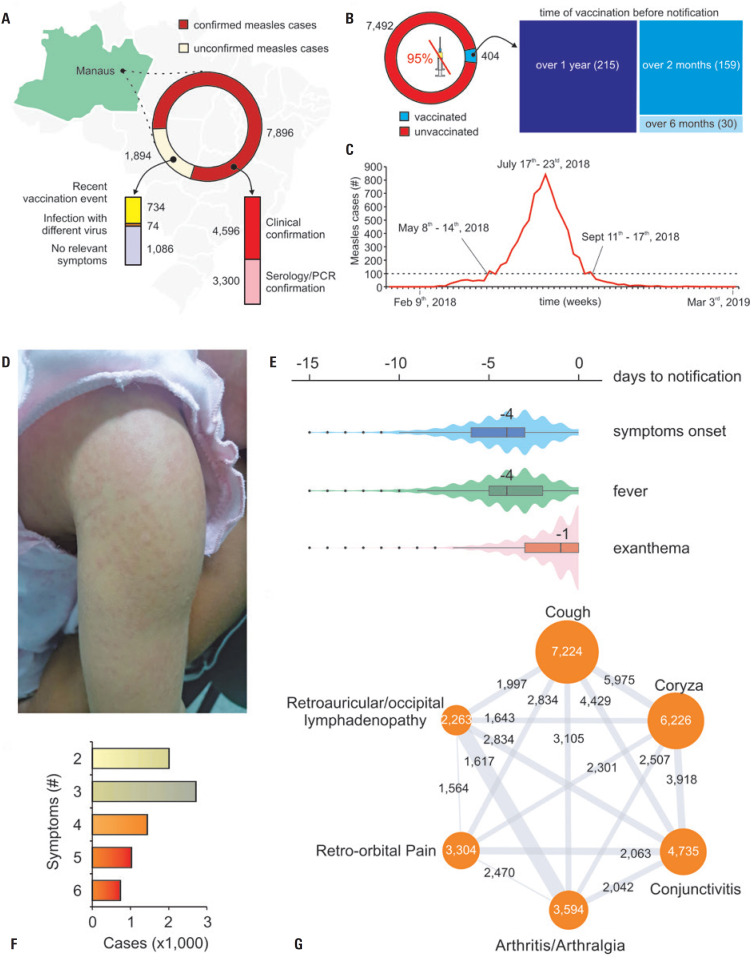
A) Map of the Brazilian states highlighting the state of Amazonas (green) and the city of Manaus (background); at the front, the circular graph shows the number of confirmed measles cases (red), with diagnostic criteria (highlighted) and unconfirmed cases (yellow) with discrimination of probable causes for misdiagnosis (highlighted below); B) Circular graph illustrating the number of vaccinated and unvaccinated individuals from the confirmed measles cases. The 818 individuals reportedly vaccinated over 10 years ago are included among the unvaccinated. Approximately 5% of the confirmed cases belonged to individuals vaccinated over different periods (in blue); C) Chart depicting the weekly number of reported measles cases during the outbreak period. The dotted line represents 100 cases, which marks the beginning and end of the outbreak with respective dates; D) Photograph depicting the characteristic exanthema (rash) of measles infection; E) Violin plot showing the median onset (of the days before notification) of general symptoms, fever, and exanthema; F) Bar plot indicating the frequency of symptoms in reported cases. Most patients exhibited only a fraction of measles-associated symptoms; G) Graph showing the co-occurrences of reported symptoms. The edges represent the number of cases presenting each particular symptom. The line numbers represent the number of cases shared between the symptoms, and the line thickness denotes the degree of association between the symptoms

The duration of each phase in the disease cycle (i.e., incubation period, symptom onset, and transmission period) may vary significantly among infected individuals. While patients typically took four days (median, IQR = 3) to report the disease after experiencing symptoms (Figure 1E), they tend to report the condition more quickly when exanthema appeared (Figures 1D and 1E) (median = 1, IQR = 3, Figure 1E). Notably, most patients reported three symptoms (Figure 1F). When comparing the co-occurrence of symptoms (Figure 1G), the strongest association was found between arthritis/arthralgia and auricular/occipital lymphadenopathy.

Demographic data were also analyzed; the most frequently diagnosed patients were males (55%; Figure 4S [A], Supplementary Material), which was not significantly different from the census data (2010), according to a previously conducted chi-square test. Regarding ethnicity, most cases involved people of color, including black-skinned people and those of mixed ancestry (any combination of black, white, and native Brazilians; Figure 4S [B]). Measles infection exhibited two age peaks: one occurring before two years of age and another at 18 years of age (Figure 4S [C]). Similarly, this age range distribution did not differ significantly from the census data. Furthermore, cases were stratified by age groups, and the reported probable locations of infection were analyzed because the exact site of infection was unknown. As expected, most children under 2 years of age were likely infected at home or in hospital settings, while teenagers (between 10 and 18 years old) were potentially infected at school (Figure 4S [D], Supplementary material). However, for young adults (aged 18 to 65 years), the probability of local infection remains unclear, as while many reported no previous contact with an infected person, most patients claimed to have been infected at work or home (Figure 4S [D], Supplementary Material).

Despite not being the focus of this study, nosocomial infections play an essential role in the introduction and spread of measles. Filia et al.^(11)^ reported an outbreak that originated in cruise ship passengers and subsequently spread via nosocomial transmission, accounting for almost 50% of the cases. They recommended that healthcare facilities ensure susceptible healthcare workers are vaccinated against measles and implement adequate infection control procedures.

While most measles cases came from a single and unique household location, almost 2,000 cases originated from over 500 households (Figure 5S [A], Supplementary Material). For instance, household location 3620 was home to five patients (Figure 5S [B], Supplementary Material). From this particular location, the second patient became aware of the disease ten days after the first patient, and the remaining patients in the household took 23 – 24 days before exhibiting signs of infection (Figure 5S [A], Supplementary Material). However, when considering all cases from individuals living in the same household, the number of days between reports varied considerably (median = 7 days; (Figure 5S [C], Supplementary Material). Notable examples include a site with numerous cases distributed over four months (Figure 5S [D], Supplementary Material) and another with nine male patients (Figure 5S [E], Supplementary Material).

For spatial analysis, we utilized patient households' geographical coordinates (latitude/longitude) to map almost 8,000 cases onto the Manaus map. A standard distance of 6.7km, representing the average distance of households from the spatial mean center, was used to perform a Kernel Density Estimation. Figure 2A illustrates a density map depicting the highest and lowest concentrations of measles in Manaus. The highest density of case households was recorded in the neighborhoods of Cidade de Deus, Jorge Teixeira, Tancredo Neves, Novo Aleixo, São José, and Zumbi. Because the concentration of residential settlements varied across neighborhoods, we calculated the infection risk percentage for each neighborhood (number of cases per neighborhood divided by the neighborhood population). Figure 2B shows the infection risk percentage map. However, the risk percentage heatmap (normalized by population) did not correlate with the Kernel Density Estimation (KDE) heatmap. Furthermore, it is important to note that neighborhoods in the east, central west, and northwest of Manaus had the lowest population densities (Figure 6S, Supplementary Material).

**Figure 2:**
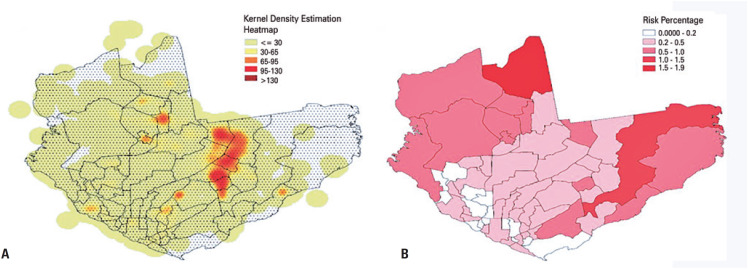
A) Density heatmap generated through Kernel density analysis of household coordinates of confirmed patients with measles; B) Heatmap depicting risk percentage calculated by dividing the number of cases per neighborhood by the neighborhood population (*i.e*., case density normalized by the population)

Next, a SIR epidemiology model was created to understand the underlying mechanisms influencing the spread of the disease and propose potential control strategies. The dataset indicated daily maximum and total infected populations of 1,576 and 7,759, respectively (Figure 3A). The estimated numbers of exposed, resistant, and infected individuals were comparable. The SIR model predicted daily maximum and total infected populations of 1,899 and 7,917, respectively (Figure 3A). Parameterization revealed that approximately 5.1% of the population remained susceptible to the disease.

**Figure 3:**
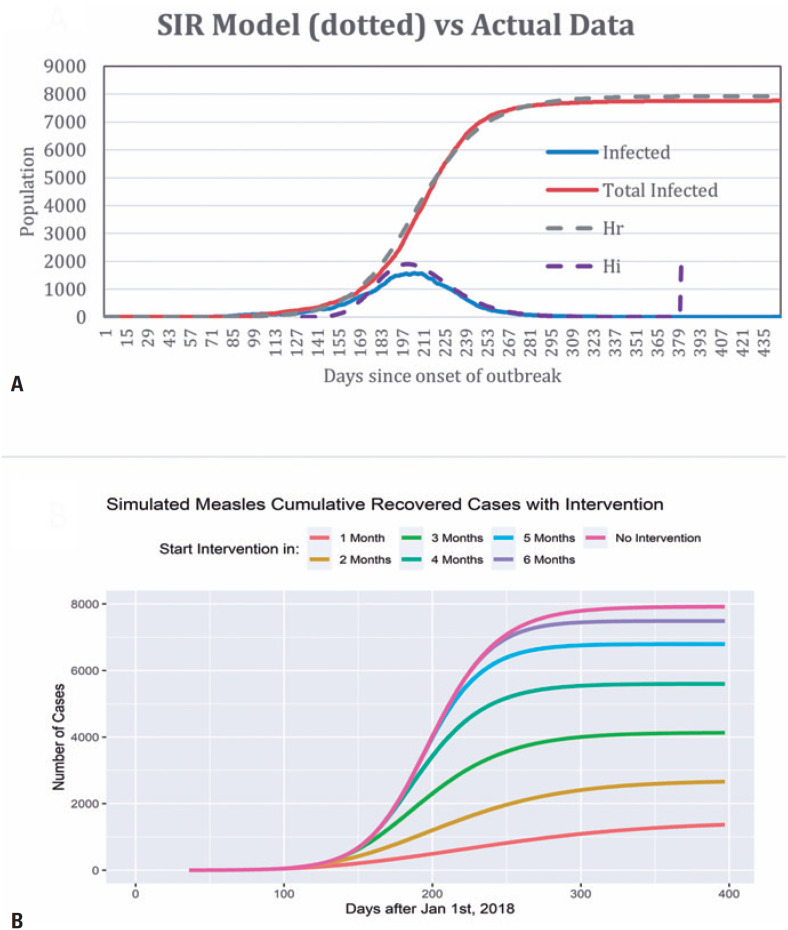
A) Chart showing the results of the SIR model against real data for the measles outbreak in Manaus. The lines show the number of individuals infected (Hi) and recovered (Hr). The total population of hosts is denoted by H, where H = Hs + Hi + Hr, with Hs representing susceptible individuals; B) The data collected is represented by solid lines. We apply dynamic contact over days after the commencement of the intervention to reflect the effectiveness relative to the intervention start time. Notably, we observed a rapid containment of 1,408 infections, representing a reduction of over 80% in infections when the intervention began a month after the first reported case

Using the estimated SIR parameters, we explored interventions to reduce infection (*e.g*., public campaigns encouraging social distancing and catch-up vaccines). Figure 3B illustrates a scenario in which dynamic contact rates were employed to reflect public action in reducing contact by 20% two months after the intervention began. Our findings showed rapid containment of 1,408 infections (representing a reduction of over 80%) when the intervention began a month after the first reported case. Moreover, each monthly delay reduced the gain by 20%. Thus, these analyses underscore the importance of timely action in achieving rapid containment and reduction of total infections.

Finally, a website was developed to facilitate the visualization of cases in the outbreak on a spatiotemporal scale. This interactive map is freely available at https://carlosprete.shinyapps.io/measles. The tool enables users to visualize all data points, which are color-coded based on the time of notification, sex, and age. There are also many other additional features, including dynamic visualization (animations) and differentiation between laboratory-confirmed and clinically confirmed cases. Figure 4 shows a screenshot of the interactive visualization tool, displaying the spatial distribution of measles cases presented as a heat map.

**Figure 4:**
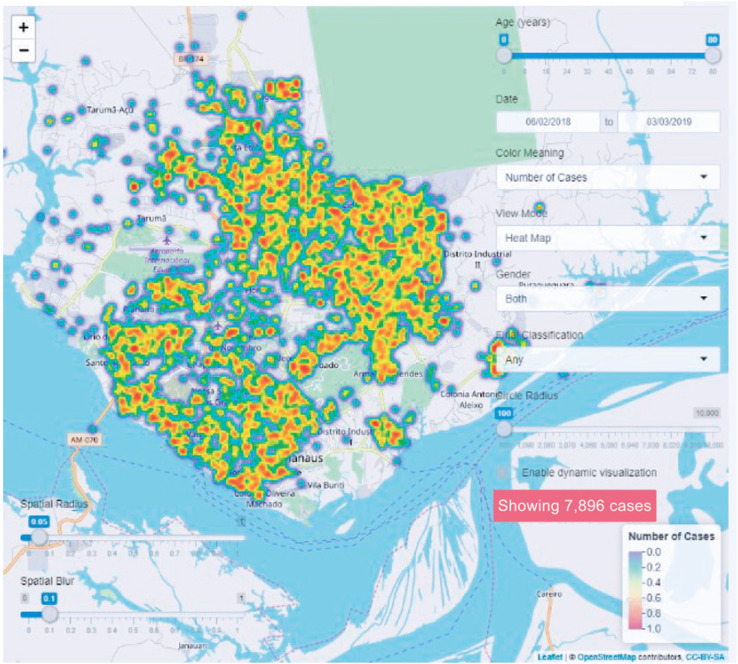
Screenshot of the interactive visualization tool displaying the spatial distribution of measles cases as a heat map

## DISCUSSION

Early details of the measles outbreak were described by Elidio et al.,^(5)^ who reported 7,602 notifications, of which only 1,631 were confirmed cases. Their study speculated that the Brazilian measles outbreak may have resulted from a previous outbreak in Venezuela due to the recent influx of immigrants from that country. However, despite the circulating virus genotype matching that in Venezuela, no Venezuelan citizens were reported to have contracted measles.

Significant variations were observed in all aspects of the disease, including the incubation period and symptomatology. Spatial modeling attempts of the outbreak revealed that the distribution of cases mirrored population density and the standard of living. Additionally, we found that the Brazilian government's investment in vaccination has declined.

Given that most of our participants were not vaccinated against measles, we hypothesize that the 5% who claimed to be vaccinated were likely unaware of the full vaccination calendar and probably did not take all the required doses for immunization. According to the Ministry of Health, the Brazilian vaccination schedule involves two doses administered between 0 – 15 months and additional doses later in life.^(12)^ Consequently, the vaccination schedule compliance by 5% of the population remains unclear, and those lacking a complete vaccination history or unable to recall receiving the vaccine should be administered the vaccine. Despite the measles vaccine being freely available at public health centers in Brazil, the Ministry of Health needs to promote awareness regarding the timely vaccination of infants. Notably, in 2017, the year before the outbreak, the federal budget for disease prevention had decreased by 33% compared to the previous year.^(13)^ Moreover, official statistics from the Brazilian Ministry of Health show a remarkable decline in vaccination coverage throughout the Brazilian territory since 2014. For instance, measles vaccination coverage in the city of Manaus alone plummeted to 67% in 2017, down from 100% in 2014.^(14)^ As a result, the 2018 measles outbreak appears to be a consequence of neglected disease prevention measures, and if this trend persists, such events may become more frequent. Importantly, despite the Ministry of Health campaigns, vaccination coverage has averaged 70% over the last five years but dropped to 40% in 2022, far below the 95% coverage target. In the Amazon States, the average coverage is even lower, at 35% and 65% for the respective periods and 2022.^(15)^

Another critical aspect highlighted in our findings is the heterogeneity observed across all aspects of the disease. Our data showed considerable variation in symptom presentation, symptom onset times, the relationship between symptoms, the interval between cases within the same household, and the participants' age. This heterogeneity significantly complicates the prediction of this disease behavior within the population. While the number of cases may fluctuate from one week to another, the substantial variance in the timing of symptom onset makes it difficult to determine infection occurrences accurately, which, in turn, complicates the identification of disease hotspots.

Regarding the geographical data of patient households, we attempted to model the spatiotemporal progression of the outbreak using the geographic coordinates of the infected households and the diagnosis dates of the notified cases. Our objective was to estimate the radius expansion of the point-containing area and utilize this information to develop action plans to prevent further case increases. However, despite employing various time-series and clustering techniques, we could not construct a model that accurately captured the spatiotemporal evolution of this disease. As a result, we limited our analysis to KDE (Figure 2A). To assess whether the kernel clustering results aligned with the demographic data, we generated a case density map normalized by the population per neighborhood (Figure 2B). This heatmap exhibited minimal overlap with the kernel density map due to variations in population density and the number of cases across neighborhoods. The neighborhoods in the municipality of Manaus, known to be among the poorest^(16)^ and most densely populated areas (Figure 3S, Supplementary Material), exhibited a higher density of cases, as shown in the Kernel Density heatmap (Figure 2A). A similar pattern was observed in the Compensa (southwest) and M. Das Oliveiras (center-north) neighborhoods (outside the concentrated density region), which also had poor living conditions and high population density (Figure 3S, Supplementary Material).^(16)^ Notably, all these areas have been identified as priority areas for leprosy prevention.^(17)^ Therefore, economic and social factors, mainly the lack of vaccination, may likely explain the distribution of measles cases during this outbreak.

The SEIR model presented in this study relies on probabilities and is influenced by factors such as the population size, initial infection rate, and contact rate. The model's performance is sensitive to multiple parameters used in its construction, and alterations in these parameters may affect the outcome. Our predicted results closely mirrored the actual data, thereby validating our parameter estimation algorithm for optimizing disease parameters and estimating susceptible population size. The intervention analysis underscores the importance of swift actions in mitigating infections to achieve rapid containment. Responding within a month could potentially reduce infections by 80%, while each subsequent month of delay diminishes this gain by 20%. Similar patterns were observed with varying contact rates. Human behavior, which is both dynamic and unpredictable, was incorporated into our modeling effort; therefore, more sophisticated analyses are necessary to understand the driving forces behind specific actions and behavior patterns.

## CONCLUSION

As demonstrated earlier, most, if not all, aspects of the disease are highly variable, and this limits predictions regarding the progression of the outbreak. Nevertheless, because the data were collected during an outbreak and in the context of health surveillance, known weaknesses must be acknowledged. Our findings indicate a higher virus spread in denser and economically disadvantaged areas. Importantly, future outbreaks will likely follow this trend, underscoring the need to prioritize these underprivileged areas and citizens for vaccination and prevention campaigns. However, measles outbreaks remain inevitable if a measles virus reaches ideal conditions, such as those observed in Manaus, a densely populated city with significant pockets of unvaccinated individuals. Vaccination remains the only way to control this disease. According to data from the Ministry of Health, emergency campaigns must focus on children aged 0 – 4 years, which means creating awareness among parents about vaccinating their children. The Brazilian Ministry of Health must intensify campaigns aimed at the general population, accompanied by increased budget allocations, to bolster measles and other infectious disease prevention efforts. Otherwise, the risk of future outbreaks persists under the current conditions.

Finally, the lessons learned from Brazil underscore the importance of high vaccination coverage to prevent the importation of measles virus from countries with active transmission, thereby averting new outbreaks. Furthermore, combating measles (and other infectious diseases) requires integrated campaigns to achieve domestic and international vaccination coverage.

## Figures and Tables

**Figure 1S:**
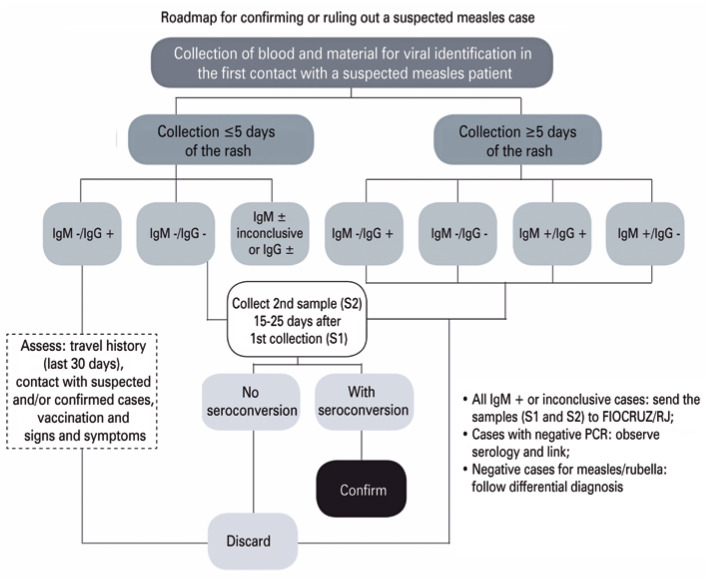
Flow chart for the confirmation of measles cases in Brazil as described by the Federal Ministry of Health

**Figure 2S:**
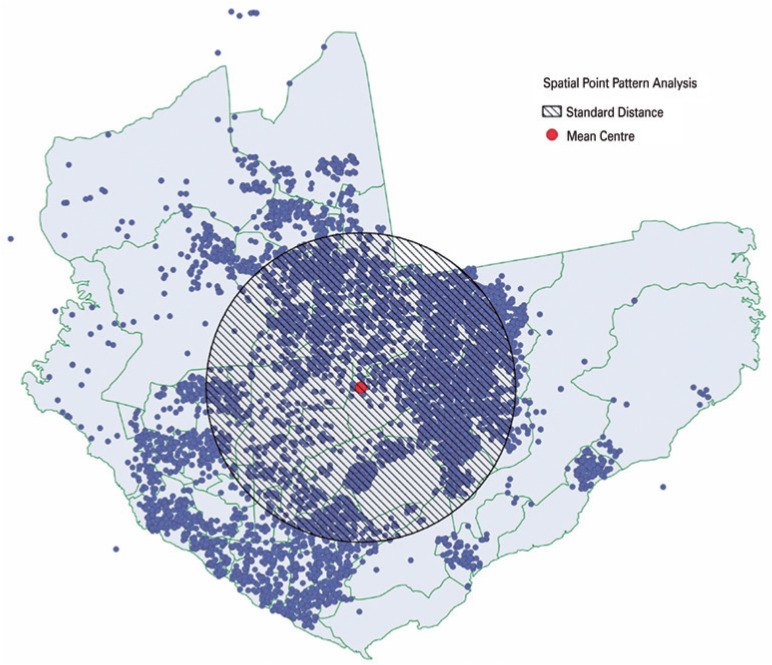
Map of the city of Manaus showing the confirmed measles cases (household points) outlined based on the neighborhood. The red dot represents the spatial mean center, while the radius of the black circle indicates the spatial standard distance

**Figure 3S:**
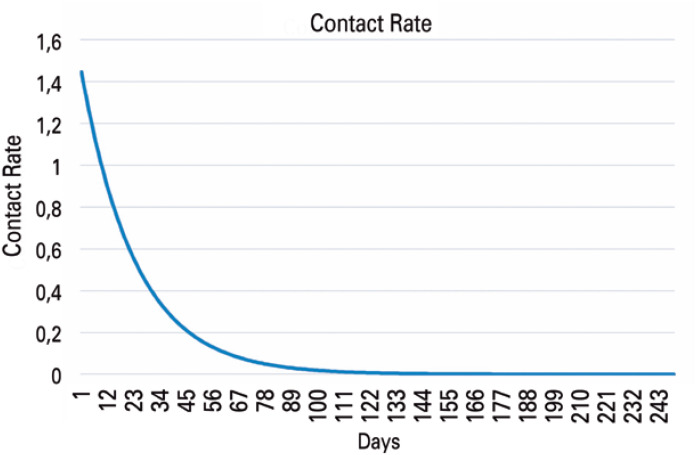
The graph shows how the contact rate changes over time

**Figure 4S:**
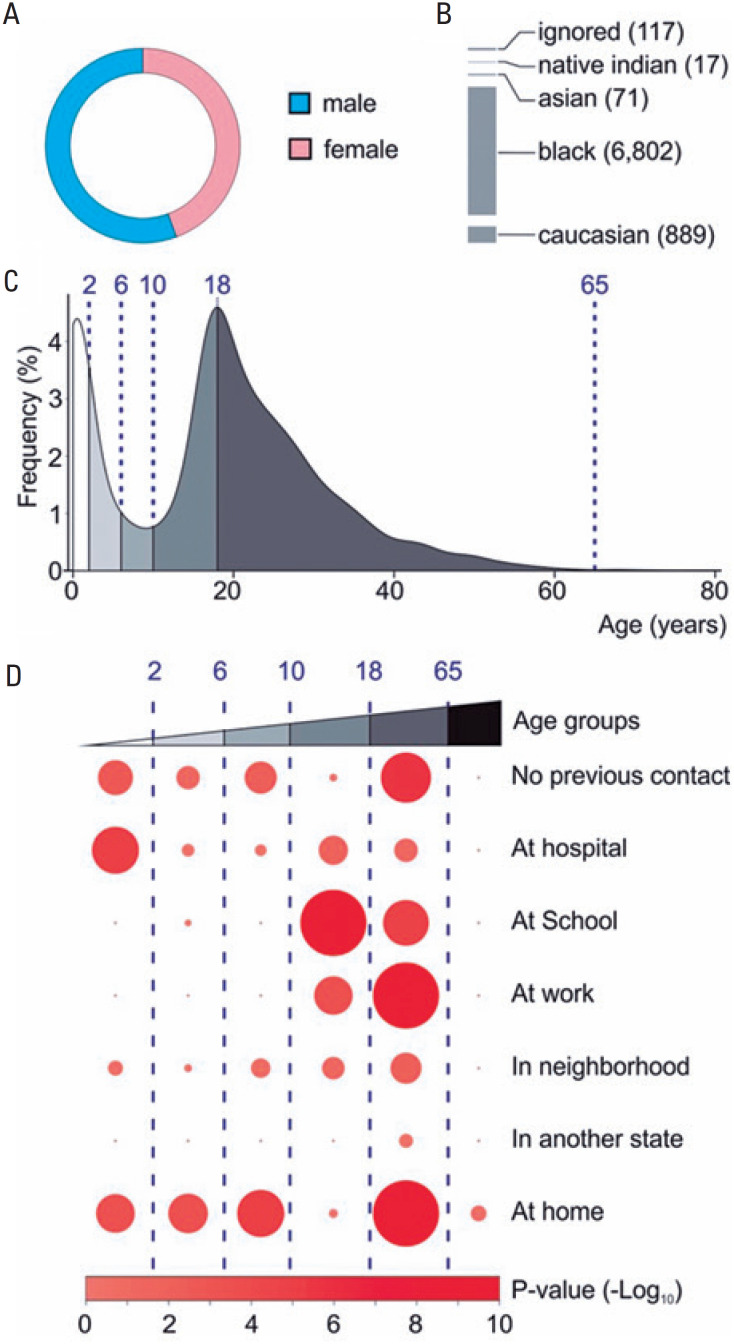
Demographics and other characteristics of the infected population. A) Proportion of male and female individuals reported with measles infection; B) Stacked bar chart indicating the number of cases shared between different ethnicities. Note: Black includes individuals of mixed ancestry ("*pardos*," literally translated as "brown-skinned"); C) The frequency distribution graph by age group. Colors are associated with age groups; D) Illustration of Fisher's exact test performed on possible sites of infection in different age groups. The size of the circle is proportional to the frequency of cases, and the color indicates the p-value

**Figure 5S:**
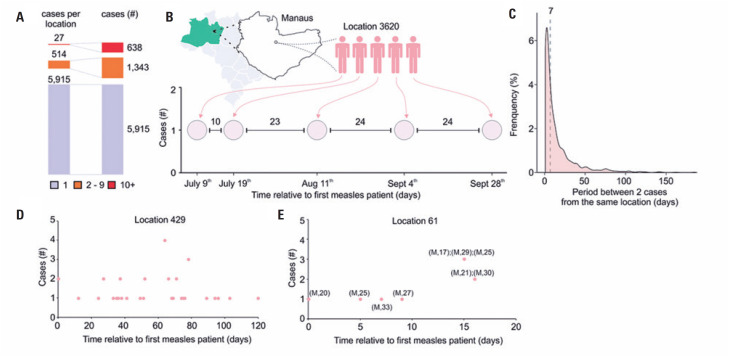
A) Stacked bar plot showing the number of cases per location and absolute number of cases associated with location class; B) A sample case showing a location with five measles cases reported and the length of time (in days) of the intervals between notifications; C) Frequency distribution of the number of days between notifications of two or more cases from the same location. The median is seven days; D) A chart showing the number of cases and the length of time (in days) from the first notification for an example location; E) A picture of the building (location 61) with nine cases reported and chart showing the number of cases and length of time (in days) from the initial notification for this same location. All cases were male patients

**Figure 6S:**
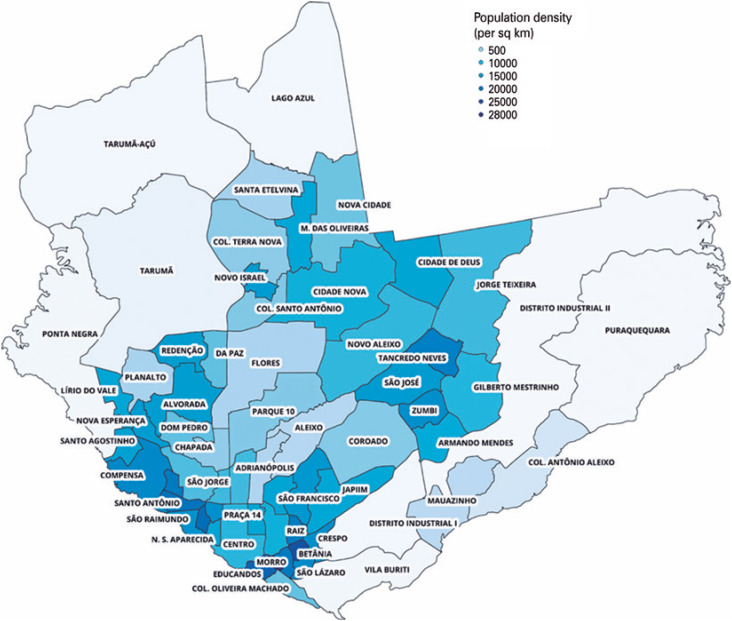
Population density of Manaus per neighborhood. The east and west zones of the Manaus region exhibit the lowest population density against the central region

## SUPPLEMENTARY MATERIAL Temporal and spatial analysis of over 7,000 measles cases outbreak from 2018 to 2019 in the Brazilian Amazon

Felipe de Mello Martins, Alessandra Pinheiro Vidal, Jeevan Giddaluru, Bernardo Maia da Silva, Eva K. Lee, Peijue Zhang, Lucas Esteves Cardozo, Carlos Augusto Prete Junior, Helves Humberto Domingues, Ester Cerdeira Sabino, Vanderson de Souza Sampaio, Wuelton Marcelo Monteiro, Helder I Nakaya

DOI: 10.31744/einstein_journal/2024AO0931

### Compartmental Epidemiology Disease Model

The compartmental system comprised four stages: *S* (susceptible), *E* (exposed), *I* (infectious), and *R* (recovered). Let *HS, HE, HI,* and *HR* denote the numbers of susceptible, exposed, infectious, and recovered individuals, respectively. Let *Hm* be individuals who have received the proper preventive measles vaccine and are thus protected from contracting the disease. The total population of *N* equals the sum of *HS, HE, HI, HR,* and *Hm.* However, in reality, some individuals may die.

Table 1S shows the parameters used in the model. The total population was 2,145,444. Because measles mostly infects children who are not vaccinated, we set the susceptible population at 1% of the total population.

**Table 1S:** Parameters and the respective values used for the epidemiological modeling

Variable name	Variable	Initial value
Human population growth rate	λ_h_	0
Human mortality rate	μ_h_	0
Contact probability	C	1.445
Inverse exposure time, human	τ_exh_	1/10 days
Inverse infective time, human	τ_ih_	1/15 days
Host resistance	R^p^	20% per 5 days

The following system of ordinary differential equations describes the SEIR model for measles:

$$\begin{array}{c} \frac{dH_{s}}{dt}=\lambda_{h}N-H_{s}\left(\frac{CH_{I}}{N}+\mu_{h}\right)\\ \frac{dH_{E}}{dt}=C\frac{H_{I}}{N_{\tau }}H_{s}-H_{E}\left(\tau_{exh}+\mu_{h}\right)\\ \frac{dH_{I}}{dt}=H_{E}\tau_{exh}-H_{I}\left(\tau_{ih}+\mu_{h}\right)\\ \frac{dH_{R}}{dt}=H_{I}\tau_{ih}-\mu_{h}H_{R}\\ \frac{dC}{dt}=-C\left(1-R^{p}\right) \end{array}$$

We designed the contact rate to be dynamic; it starts at a high value and decreases by 20% every five days to emulate participants' behavior changes as they become aware of the outbreak.
